# CID fragment annotation from data-independent experiments in non-target organic aerosol analysis: presenting an easy-to-use tool

**DOI:** 10.1007/s00216-025-06184-5

**Published:** 2025-11-18

**Authors:** Niklas Karbach, Thorsten Hoffmann

**Affiliations:** Johannes-Gutenberg University Mainz, 55128 Mainz, Germany

**Keywords:** CID fragment identification, LC/HRMS, Organic aerosol, Non-target analysis

## Abstract

The open-source tool presented in this work provides a simple, reliable, and comprehensive method for analyzing and annotating CID fragments of precursor ions in data-independent full MS/AIF experiments in non-targeted HPLC-UHRMS analyses (NTA). The method provides similar results to established data-dependent fragmentation experiments while simultaneously increasing the informational value of the resulting raw data file, giving a more unbiased view of the sample, which is crucial for NTA, and improving the limit of detection for fragment ions. Using the tool to analyze a mixture of 28 standard compounds has shown that fragment identification and annotation were equal to or better for 71% of the measured compounds — compared to manual analysis — while being completely automated and not requiring input from the experimenter. Formula prediction performed better, with the tool being able to correctly identify 96% of the standard compounds. The purpose of this paper is to provide an easy-to-use tool to facilitate the usage of full MS/AIF measurements for non-targeted analysis, automating the process of fragment annotation and identification for structural analysis of precursor ions. The proposed workflow will significantly increase the informational value of each measurement file, allowing for the creation of databases, and ultimately speed up scientific progress. The algorithm presented in this work yields comparable results as would be obtained by manual analysis of isolated MS^2^ spectra.

**Graphical Abstract:**

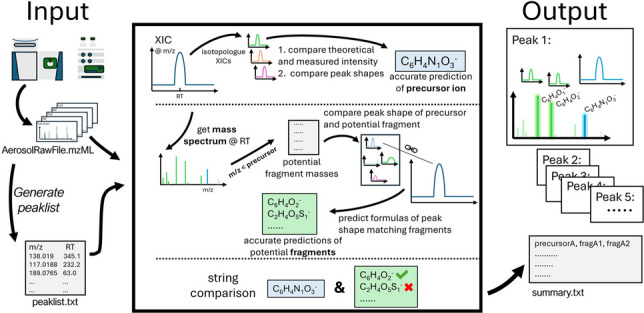

**Supplementary Information:**

The online version contains supplementary material available at 10.1007/s00216-025-06184-5.

## Introduction

Organic particulate matter makes up a majority of total atmospheric aerosol (OA) mass [1–3]. Studies have shown that OA contains thousands of different species of different compound classes with different functionalities, polarities, and structures [4–6]. Untargeted UHPLC/HRMS analysis with a soft ionization technique (e.g., ESI) can separate those compounds and capture their exact masses [7]. When simultaneously performing fragmentation experiments, information about the structure of the compounds contained in the sample can be extracted [8]. This approach allows for a comprehensive investigation of the complex mixtures that are contained in the sample. Information about the organic compounds provides insights into sources [9, 10], transformations [11], and impacts on climate and air quality [3, 12]. However, the amount of data generated from such non-targeted analyses is considerably higher than for targeted experiments, so manual evaluation of the results is not applicable anymore.

Previously, a large number of non-targeted LC/MS analyses about OA was conducted with only full MS measurements providing only information about the compounds contained in the sample, but not about their structure [13–15]. On the other hand, fragmentation experiments dissociate the molecular ions by collision with an inert collision gas, generating a fragment ion and a corresponding neutral loss, of which the fragment ion can be measured in the MS. This dissociation is dependent on the stability of the precursor ion and therefore dependent on the connectivity and (sub-)structure of the molecule. The fragmentation pattern can be used to extract structural information about the molecular ion [16]. Therefore, fragmentation experiments are recently being integrated into the analysis to enhance the understanding of molecular compositions, sources, and transformations. These experiments are usually conducted using either an inclusion list that targets specific precursor ions [17, 18] or through data-dependent (e.g., dd-MS^2^) experiments, which provide a broader collection of fragmentation data [19–22]. However, to capture this information in a *truly* non-targeted approach, one must use data-*in*dependent acquisition (DIA) fragmentation experiments like all-ion-fragmentation (AIF). In this type of experiment, no inclusion list whatsoever is used that would restrict or bias the experiment or the results in any way, but rather, all ions that are produced in the source are fragmented in a single step without any preselection of defined mass-to-charge ratios. Due to the large number of fragments, manual analysis of this type of data is not applicable anymore [23–26]. *But* the generated full MS/AIF results file contains all the information that is present in the sample and therefore resembles a near-complete digital copy of the sample which can be easily stored, analyzed, and even distributed around the globe to enhance exchange between researchers to ultimately improve scientific progress.

### CID fragmentation

In a collision induced dissociation (CID) experiment, the precursor ions are trapped in a collision cell where they are colliding with an inert gas like N_2_ or He. Through these collisions, the ions are vibrationally excited, which, depending on the structure of the ion, leads to cleavage (fragmentation) of bonds in the molecular ion [27]. The resulting fragments are characteristic of molecular structures and the fragmentation energy that was used for the CID experiment. With increasing fragmentation energies, more stable bonds start to break, resulting in more (and usually smaller) fragment ions. Electrospray ionization (ESI) is particularly well suited for CID experiments, as a soft ionization source reduces in-source fragmentation, and the compounds contained in the sample are usually left intact, which ensures well-defined precursor ions for the fragmentation experiment [16].

The orbitrap provides two main types of acquisition modes for fragmentation experiments — data-dependent acquisition (DDA) and data-independent acquisition (DIA). For the DDA, the orbitrap automatically selects the most abundant precursor ions from the MS^1^ scan for an additional (isolated) fragmentation experiment [8]. This type of experiment provides clean spectra of selected precursor ions, resulting in easier manual annotation and interpretation. However, this type of analysis is significantly biased towards high-intensity compounds. Low-intensity compounds might not be selected for fragmentation. Another DDA method is to manually create an inclusion list to specify which compounds should be fragmented. As this inclusion list is created by the experimenter, it might also be biased, as unexpected compounds are not considered. DIA, on the other hand, is fragmenting all precursor ions, independent of their intensity, m/z value, or their retention behavior. This provides a comprehensive measurement of all precursor ions that are contained in the sample. In a so-called full MS/AIF experiment, the orbitrap alternates between a full MS (MS^1^) measurement without fragmentation and the data-independent MS^2^ measurement, where all ions are fragmented. This reduces the time resolution of the experiment by a factor of 2; however, it also increases the informational value of the resulting data file significantly [8].

As stated above, the fragmentation pattern of precursor ions can give information about structural elements contained in the molecule. Typically, neutral losses are used for this type of analysis, as functional groups usually have a specific neutral loss. A very typical neutral loss for molecules that have a methoxy group is CH_3_OH. Investigations have shown that if a molecular ion has CH_3_OH as a neutral loss to a corresponding fragment, the chance of the precursor ion having a methoxy group is 95.6% [28]. As Ma et al. investigated other neutral losses and their corresponding sensitivity and specificity as well [28], it becomes evident that by utilizing fragmentation information, functional groups can be determined relatively easy.

### Identification/annotation of CID fragments

Identifying CID fragments from DIA experiments is currently an emerging topic, and not much work has been done in the field of organic aerosol analysis. This is where this work focuses on.

As a lot of information is contained in the raw data file of full MS/AIF experiments, extracting useful information proves challenging, and automation is needed to process the data. Fragments of precursor ions can be identified using a two-step process. Firstly, by comparing the similarity of the retention behavior of the potential fragment and the precursor ion, and secondly by checking if the predicted sum formula of the fragment is part of the formula of the precursor ion.

Two types of spectra are available in full MS/AIF data, of which the retention behavior of the *precursor ion* is best extracted from the full MS spectra, as here no fragmentation occurred. To extract the retention behavior of the *fragment*, the AIF spectra should be used. The extracted ion chromatograms (XICs) of both ions should look similar as fragmentation occurs only after the column and is therefore not affected by the chromatographic method. A method to perform this similarity check automatically is to calculate the area between the two normalized XICs and compare the result to a given threshold value.

Another measure to automatically verify if a given signal can be a fragment of a selected precursor ion is by comparing their predicted sum formulas. The sum formula of the fragment needs to be part of the precursor ion. If, for example, the fragment contains an element or formula pattern that is *not* contained in the precursor ion, the fragment can*not* be a true fragment of the selected precursor. Here it should be noted that due to the presence of reactive oxygen species in the ESI source, in-source oxidation of analyte molecules might happen in significant quantities to form oxygenated or even double oxygenated ions [29, 30]. Those oxygenated precursor ions are then also contained in the MS^2^ spectrum and might interfere with the sum formula comparison. With the identified fragments, a quasi-isolated MS^2^ spectrum can be created which resembles the same type of spectrum as would be obtained from a DDA and thus is as easily human-interpretable as the original would be. In metabolomics/proteomics this type of DIA experiment is used more frequently [23–26]; however, despite the obvious advantages, no information about the application of DIA experiments in aerosol analysis has been found.

In this paper we propose a workflow to maximize the informational value contained in the raw data file by conducting data-*in*dependent full MS/AIF measurements. This measurement procedure does not make any restrictions during measurement; therefore, all information that is contained in the sample is also contained in the raw data file. This digital copy of the sample can then be archived in a database, allowing for the reanalysis of the sample again and again. As the complexity of an AIF spectrum is significantly higher compared to an isolated MS^2^ spectrum, manual analysis of the data is not feasible. Hence, there is a need for a tool to automatically analyze the measurements and annotate fragment ions. The tool has been developed in this work and is maintained at the following GitHub repository: https://github.com/NKa1409/UVenture.

## Methods/experimental

### Experimental plan

**Fig. 1:**
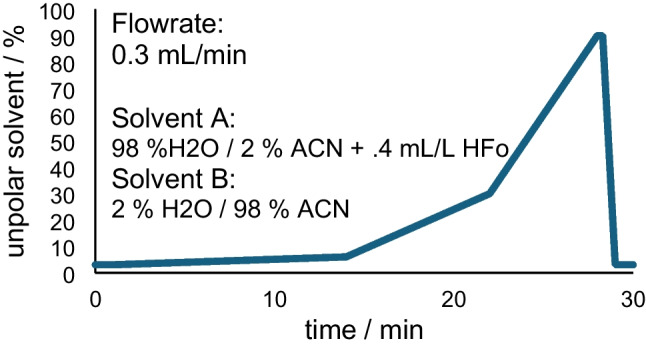
Plot of the solvent gradient used for the LC run

To evaluate the algorithm presented in this work, a standard mixture containing 28 compounds that are ionizable with ESI-(-) was measured with HPLC-ESI(-)-HRMS. The HPLC method was a standard method with a duration of 30 min and increasing ACN mixing ratio (see Fig. 1). The MS method was a DIA experiment (full MS/AIF). The results were then automatically evaluated using the tool presented in this work to yield formula predictions for the molecular ion, annotation and identification of the corresponding CID fragments, and creation of quasi-isolated MS^2^ spectra for all specified compounds.

To verify the results, a DDA experiment (full MS/dd-MS^2^ with multiplexing of up to three different masses) was conducted that measured the same standard mixture and employed the same HPLC method, but changed the MS acquisition method. These results were used to identify CID fragments of the standard compounds from a DDA experiment.

### Chemicals

A summary of the chemicals used, their quality, and the manufacturer can be found in Table 1.

The standard mixture was prepared in a 1:1 (v/v) mixture of ACN:H_2_O, and each standard had a final concentration of 1000 ppb (w/w). Ultrapure water with 18.2 MΩ resistance was produced using a Milli-Q water system from Merck Millipore. Ultrapure acetonitrile (ACN, LC/MS grade) was obtained from VWR Chemicals.

Solvents used as HPLC solvents were water (LC/MS grade) and methanol (LC/MS grade), both from Fisher Scientific, and ultrapure acetonitrile (ACN, LC/MS grade) and formic acid (LC/MS grade) from VWR Chemicals. Ammonium hydroxide solution (NH_3_, analytical grade, 25%) was from Honeywell Fluka.

**Table 1:** Chemicals used in the standard mixture

Ibuprofen (99%, Acros Organics)	2-Nitrophenol (98%, Alfa Aesar)	Salicylic acid (99%, Sigma Aldrich)
3-Maleimidopropionic acid (97%, TCI)	4-Methyl-2-nitrophenol (99%, Sigma Aldrich)	2-Sulfobenzoic acid (95%, Sigma Aldrich)
Maleic acid (> 99%, Sigma-Aldrich)	3-Nitrosalicylic acid (99%, Acros)	Syringic acid (95%, Sigma Aldrich)
2-Methylsuccinic acid (98%, BLDpharm)	2,6-Dimethyl-4-Nitrophenol (98%, Sigma Aldrich)	Syringaldehyde (98%, Sigma Aldrich)
4-Nitro-1-Naphthol (> 98%, TCI)	4-Nitrocatechol (96%, Sigma Aldrich)	Vanillin (99%, Acros)
2-Nitrobenzoic acid (95%, Sigma Aldrich)	Acetylsalicylic acid (99+%, Sigma Aldrich)	Benzoeic acid (99.5%, Acros)
Nitrobenzene (99%, Sigma Aldrich)	Pimelic acid (98%, Sigma Aldrich)	Trihydroxybenzene (97%, Sigma Aldrich)
4-Acetamidophenol (98%, Acros)	2-Carboxybenzaldehyde (97%, Sigma Aldrich)	Camphor-10-sulfonic acid (99%, Sigma Aldrich)
3′,5′-Dimethoxy-4′-hydroxyacetophenone (97%, Sigma Aldrich)	Glutaric acid (99%, Acros)	2-Iodobenzoic acid (98%, Sigma Aldrich)
Trans-Cinnamic acid (99%, Sigma Aldrich)		

### Instrument method

#### HPLC method

Separation was performed on a Dionex UltiMate 3000 UHPLC system coupled to a heated ESI source and a Q Exactive Orbitrap (Thermo Fisher Scientific). All MS spectra were acquired with a resolution of 140,000 between m/z ratios of 50–400. Two sets of fragmentation energies were used for fragmentation (a) = 11, 25, 35 (b) = 11, 30, 90. As column, an Acquity UPLC CSH Fluoro Phenyl (PFP) column, 100 mm × 2.1 mm with 1.7 μm particle size (Waters), located in a 30 °C column oven was used. For all experiments, a post-column flow of 0.05 mL/min methanolic ammonia (50 mM) was used to enhance ionization in the negative mode. ESI settings were the same as reported in [46].

A H_2_O/ACN gradient (flowrate 0.3 mL/min) was used to improve separation. The gradient used in this work can be seen in Fig. 1.

## Results

### Algorithm and the tool

The source code for the tool and the algorithm to perform the analysis is provided and maintained at the following Github repository where upcoming versions will also be posted: https://github.com/NKa1409/UVenture.

**Fig. 2:**
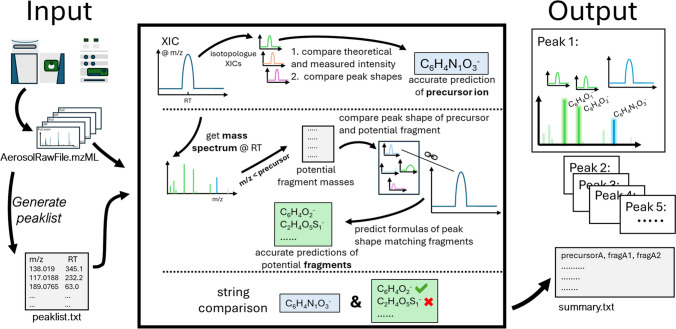
Schematic representation of the analysis workflow. After the LC-HRMS measurement, a peak list is created, which is then transferred to the tool together with the raw data file. The tool automatically processes the peak list and generates a summary file with the individual results, which can then be used for further analysis

The schematic in Fig. 2 shows the most important steps of the analysis workflow and explains the principles of the presented tool.

### Standard measurements

A standard mixture of 28 different compounds (c = 1000 ppb) was measured. The compounds were randomly selected; however, attention was paid that the compounds were ionizable with an ESI-(-) source. The algorithm was then used to predict the sum formulas of the molecular ions and annotate the fragments of each molecule contained in the standard mixture. The results of this test are summarized in Table 2. The results can be clustered into different cases.

The case where the algorithm predicted the exact same fragments as could be identified in the dd-MS^2^ spectrum accounts for 4 of the 28 compounds. The case where the algorithm predicted the same, plus additional plausible fragments, accounts for 12 of the 28 compounds. 4 compounds were correctly identified by the algorithm but did not show any fragments, both in the dd-MS^2^ spectrum as well as in the AIF spectrum. Those three cases combined account for 20 of the 28 compounds (71%). All the above-mentioned cases give either equal or better results than the evaluation of the isolated dd-MS^2^ spectra.

For the remaining 8 compounds (29%), the algorithm was either only able to find some of the fragments that were identified in the dd-MS^2^ spectra (3 compounds), or it did not find any fragments at all (4 compounds), or it did not predict the formula of the compound itself (1 compound). However, it must be noted that in no case did the algorithm predict a fragment formula that was wrong and did not belong to the precursor ion.

The total number of fragments across all compounds that were identified with the algorithm sums to 52, whereas the number of fragments identified with the dd-MS^2^ spectra only sums to 39. Note that for 3′,5′-Dimethoxy-4′-hydroxyacetophenone and trans-Cinnamic acid, the orbitrap decided not to capture a dd-MS^2^ spectrum, probably because other m/z ratios showed higher intensities at that moment.

**Table 2:** Results of the 28 standards that were measured. Two types of stepped collision energies were used to investigate the influence. The last two columns contain the fragments that were found by searching the dd-MS^2^ spectra and that were automatically identified by the algorithm

s-N(CE)	Compound	m/z	MI predicted	Fragments reference	Fragments AIF
11, 30, 90	Ibuprofen	205.1234	C_13_H_17_O_2_	x	x
11, 30, 90	3-Maleimidopropionic acid	168.0302	C_7_H_6_NO_4_	C_4_H_2_N_1_O_2_	x
11, 30, 90	Maleic Acid	115.0036	C_4_H_3_O_4_	C_3_H_3_O_2_	C_3_H_3_O_2_
11, 30, 90	2-Methylsuccinic acid	131.0349	C_5_H_7_O_4_	C_4_H_7_O_2_	C_4_H_7_O_2_, C_4_H_5_O_1_
11, 30, 90	4-Nitro-1-naphthol	188.0352	C_10_H_6_N_1_O_3_	C_10_H_6_O_2_	C_10_H_6_O_2_, C_9_H_5_O_2_
11, 30, 90	2-Nitrobenzoic acid	166.0145	C_7_H_4_N_1_O_4_	x	C_6_H_4_N_1_O_2_
11, 30, 90	2-Nitrophenol	138.0196	C_6_H_4_N_1_O_3_	C_6_H_5_N_1_O_1_	x
11, 30, 90	4-Methyl-2-nitrophenol	152.0352	C_7_H_6_N_1_O_3_	C_7_H_6_O_2_, C_6_H_5_O_1_	C_7_H_6_O_2_, C_6_H_5_O_1_, C_7_H_5_O_2_
11, 30, 90	3-Nitrosalicylic acid	182.0094	C_7_H_4_N_1_O_5_	C_6_H_4_N_1_O_3_	C_6_H_4_N_1_O_3_, C_6_H_4_O_2_
11, 30, 90	2,6-Dimethyl-4-nitrophenol	166.0509	C_8_H_8_N_1_O_3_	C_8_H_8_O_2_, C_7_H_7_O_1_	C_8_H_8_O_2_, C_8_H_7_O_2_, C_7_H_7_O_1_
11, 30, 90	4-Nitrocatechol	154.0145	C_6_H_4_N_1_O_4_	C_6_H_4_O_3_, C_5_H_3_O_2_	x
11, 30, 90	Salicylic acid	137.0244	C_7_H_5_O_3_	C_6_H_5_O_1_, C_5_H_5_	C_6_H_5_O_1_, C_5_H_5_
11, 30, 90	2-Sulfobenzoic acid	200.9858	C_7_H_5_O_5_S_1_	x	O_3_S_1_, C_6_H_5_O_3_S_1_
11, 30, 90	Syringic acid	197.0455	C_9_H_9_O_5_	x	x
11, 30, 90	Syringaldehyde	181.0506	C_9_H_9_O_4_	C_8_H_6_O_4_, C_7_H_3_O_4_, C_6_H_3_O_3_, C_5_H_3_O_2_, C_4_H_3_O_1_	C_8_H_6_O_4_, C_4_H_3_O_1_
11, 30, 90	Vanillin	151.0400	C_8_H_7_O_3_	C_7_H_4_O_3_, C_6_H_4_O_2_	C7H4O3
11, 30, 90	Nitrobenzene	122.0247	C_6_H_4_N_1_O_2_	x	x
11, 25, 35	Acetylsalicylic acid	179.0350	C_9_H_7_O_4_	C_6_H_5_O_1_, C_7_H_5_O_3_	C_6_H_5_O_1_, C_7_H_5_O_3_
11, 25, 35	Trihydroxybenzene	125.0244	C_6_H_5_O_3_	C_3_H_5_O_1_, C_5_H_3_O_3_, C_5_H_3_O_2_, C_4_H_3_O_2_	C_3_H_5_O_1_, C_5_H_3_O_3_, C_4_H_3_O_3_
11, 25, 35	4-Acetamidophenol	150.0561	C_8_H_8_N_1_O_2_	C_6_H_5_N_1_O_1_, C_4_H_4_N_1_O_1_, C_6_H_4_N_1_O_2_, C_6_H_6_N_1_O_1_, C_5_H_4_N_1_O_2_	C_6_H_5_N_1_O_1_, C_4_H_4_N_1_O_1_, C_7_H_4_N_1_O_2_, C_6_H_4_N_1_O_2_, C_5_H_4_N_1_O_1_, C_6_H_6_N_1_O_2_, C_6_H_6_N_1_O_1_, C_5_H_4_N_1_O_2_, C_7_H_6_N_1_O_2_
11, 25, 35	Pimelic acid	159.0663	C_7_H_11_O_4_	C_6_H_11_O_2_, C_6_H_9_O_1_, C_6_H_7_O_1_	C_6_H_9_O_1_, C_6_H_11_O_2_, C_6_H_7_O_1_, C_2_H_3_O_3_, C_6_H_9_O_2_, C_7_H_9_O_3_
11, 25, 35	Benzoeic acid	121.0295	C_7_H_5_O_2_	x	C_7_H_5_O_1_, C_6_H_5_O_1_, C_6_H_5_
11, 25, 35	3′,5′-Dimethoxy-4′-hydroxyacetophenone	195.0663	C_10_H_11_O_4_	NO MS^2^	x
11, 25, 35	2-Carboxybenzaldehyde	149.0244	C_8_H_5_O_3_	C_7_H_5_O_2_, C_7_H_5_O_1_	C_7_H_5_O_2_, C_7_H_5_O_1_, C_2_H_3_O_3_, C_6_H_5_O_1_, C_6_H_5_
11, 25, 35	Camphor-10-sulfonic acid	231.0697	C_10_H_15_O_4_S_1_	O_3_S_1_	O_3_S_1_
11, 25, 35	trans-Cinnamic acid	147.0452	NO FORMULA	NO MS^2^	x
11, 25, 35	Glutaric acid	131.0350	C_5_H_7_O_4_	C_4_H_7_O_2_	C_4_H_7_O_2_, C_4_H_5_O_1_
11, 25, 35	2-Iodbenzoic acid	246.9262	C_7_H_4_I_1_O_2_	I	x

## Discussion

The results showed that the annotation algorithm for CID fragments provided good results without manual input from the experimenter. For 20 of the 28 compounds (71%), the algorithm either performed equally well or even better at finding matching fragment ions than the evaluation of the dd-MS^2^ spectra. For only 8 of the 28 compounds, the algorithm found fewer or no fragments than could be identified in the dd-MS^2^ spectra, or it did not predict the formula of the compound itself. These results clearly show that the annotation algorithm is suitable for replacing traditional DDA experiments with DIA experiments. In combination with the more unbiased view on the sample and the generally higher intensity of fragments during DIA experiments, the proposed method provides significant advantages over DDA experiments.

For 12 of the 28 measured compounds, the algorithm did find more fragments than were present in the dd-MS^2^ spectrum. This can easily be explained by the significantly lower abundance of the precursor ion in isolated MS^2^ experiments than in AIF experiments (see Fig. 4 and Fig. 5). This phenomenon is another huge advantage of the proposed workflow, as more fragments, and therefore more structural information, can be extracted from the measurements.

It must be mentioned that the evaluation of the full MS/AIF data was done completely automated without any user input, whereas the analysis of dd-MS^2^ spectra was a tedious work of manually checking all dd-MS^2^ spectra for fragments of the precursor ions. This clearly shows a significant advantage of the tool over manual analysis approaches, as despite the significantly faster and completely automated analysis, the results are still comparable.

### CID fragment annotation from data-independent experiments

The workflow that is used by the tool for detecting and annotating CID fragments of selected parent ions is a two-step process of first comparing the retention behavior of the selected precursor ion to all possible fragment masses. If the area between the parent XIC and the fragment XIC is below a defined threshold value (see Fig. 3), the fragment is considered in further processing. In a second step, the sum formula of the fragment is determined and then compared to the sum formula of the parent ion. If the fragment contains any elements or atom patterns that are not part of the parent ion, the specific fragment cannot be a true fragment of the selected precursor ion and is therefore not considered. Although it is sometimes used and might improve prediction results, it was chosen not to implement a database search, as this would lead to biased results. Instead, all sum formulas and fragments should be considered equally.

To get reliable fragment annotation, both of these steps need to provide good results individually. Therefore, a lot of development work has gone into adjusting both algorithms individually before finally combining them in the finished tool. The following chapters will give an overview of the development process and its findings.

1. CID fragment masses for a corresponding precursor ion are detected based on the peak shape similarity between the given precursor ion mass and the potential fragment mass. To get the highest intensity fragment XIC the tool uses the available AIF spectra. For the precursor ions the tool selects the available full MS spectra for the same reason. Depending on the noise level of the XIC, a threshold value for the area between the curves is set, below which the potential fragment mass is considered for further processing. If the calculated area between the curves is higher than said threshold, the fragment mass is not considered for further processing (see Fig. 3). By not considering the mass in further computations, a lot of computing power (and therefore time) can be saved without influencing the results.

**Fig. 3:**
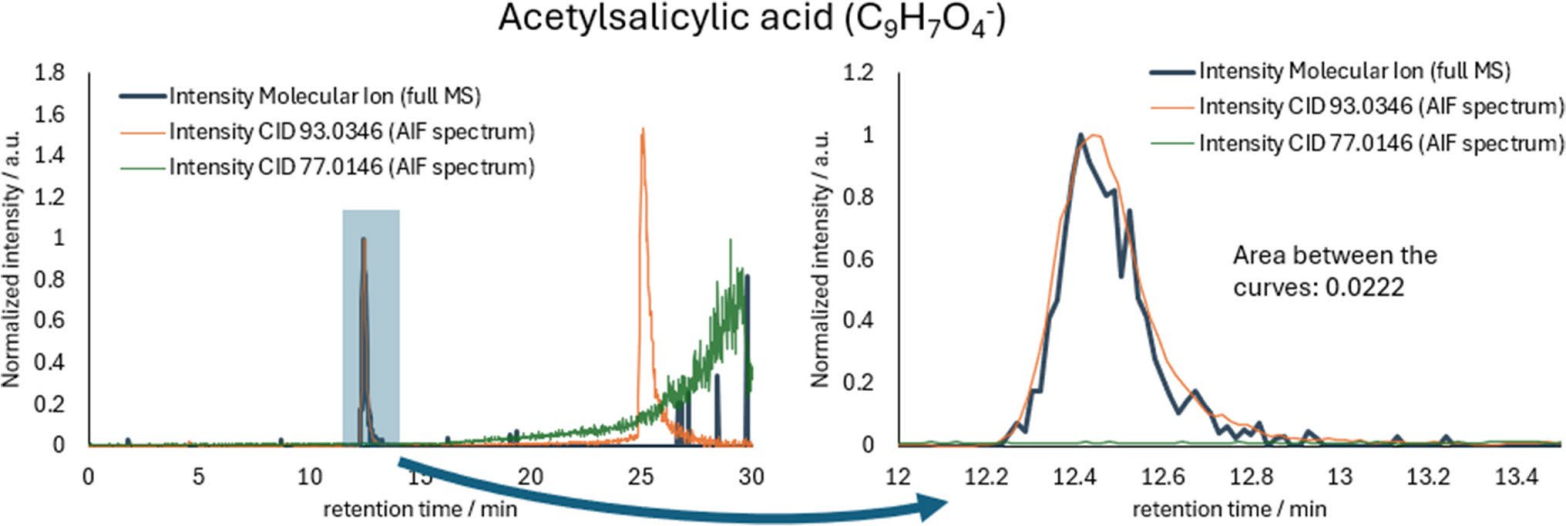
(**left**): Overlaid normalized XICs for acetylsalicylic acid (precursor ion; m/z = 179.0351; blue line), the related fragment with m/z = 93.0346 (C_6_H_5_O_1_; neutral loss = C_3_H_2_O_3_; orange line) and the unrelated CID fragment m/z = 77.0146 (green line). The fragment XICs were constructed using the MS^2^ spectra. (**right, zoomed in XIC**): It is visible that the retention behavior of the precursor ion and the fragment ion (m/z = 93.0346) is the same, indicating that m/z = 93.0346 is a true fragment, whereas m/z = 77.0146 is unrelated to the precursor ion
2. Once potential fragment *masses* have been identified, the sum formula of these fragments must be predicted. For this, the algorithm uses isotopologue pattern matching to find the most probable molecular formula. The measured isotopologue pattern is compared with the theoretical pattern of all formulas which have a mass deviation of ±15 ppm from the selected fragment mass. Additionally to comparing the intensities, the normalized XIC of the isotopologue masses is calculated and compared against the peak shape of the precursor ion, where again the area between the curves is calculated as a quality measure. A scoring function then takes all the inputs like the deviation of the measured mass to the theoretical mass of the molecule, the isotopologue matching, and the similarity of the XIC peak shapes, and calculates a score for each possible molecular formula.
  The algorithm is designed to reject a potential formula, if no second most intense isotopologue signal can be found. This feature helps significantly reducing the number of false positives. However, as the second most abundant isotopologue usually has a significantly lower intensity than the most abundant, prediction of low intensity signals is sometimes not possible. In this case, using the exact mass alone would be the only option to make a prediction about the sum formula of the selected ion. During development, it could be seen that using the exact mass alone did not provide satisfactory results as the number of false positives hugely increased.
  The likelihood of a chemical formula (ring and double bond equivalents (RDBE), carbon to hydrogen ratio (C/H), phosphor to oxygen ratio (P/O), …) can optionally be used to increase reliability of the prediction. This is achieved by introducing a punishment score that punishes unlikely elemental compositions which is then also considered in the scoring function.
  As can be seen, for 27 of the 28 compounds the correct formula could be identified and not a single false positive formula was predicted, indicating that this process gives reliable results, especially if the ion abundances are high. For low intensity signals where the second most abundant isotopologue signal could not be found, the algorithm does not provide a prediction at all, which helps to decrease the number of false positives and therefore increases the trustability of the results.

### Comparison with data-dependent experiments

In comparison with traditional DDA experiments, the workflow presented in this work poses three main advantages. The first advantage is that the measurement results are not biased in any way, either by the instrument or the experimenter itself, so all information that is contained in the sample is also contained in the raw data file. The second advantage is that the construction of quasi-isolated MS^2^ spectra is proven to be possible, and therefore the easy human readability of the generated MS^2^ spectra is maintained. The third advantage is that in the AIF spectrum, more fragments can be identified than in the dd-MS^2^ spectrum, as the total intensity of the AIF spectrum is significantly higher than that of the isolated MS^2^ spectrum.

A disadvantage of DIA experiments is that coelution of species might lead to problems if the retention behavior of both compounds, and their sum formulas are very similar, as then both of the processes mentioned in the “CID fragment annotation from data-independent experiments” section would fail to separate the fragments of the two precursor ions.

#### Truly unbiased workflow

In contrast to traditional DDA experiments (e.g., dd-MS^2^ (Top N) or with an inclusion list), the results of a full MS/AIF experiment are not biased in any way. This means that all information contained in the sample is captured, and no selection by the experimenter or the instrument is made. In fact, during this work, for 2 of the 28 compounds (3′,5′-Dimethoxy-4′-hydroxyacetophenone and trans-Cinnamic acid), the instrument decided to not conduct a dd-MS^2^ experiment — most probably as their intensity was too low compared to the other ions that were being measured at that time. In a real sample, this information would be lost, whereas in the full MS/AIF data, this information is still contained, although the algorithm is currently not yet powerful enough to extract this information.

The proposed workflow is also unbiased in the way that no database is used to interpret the fragmentation pattern or help with formula prediction. Especially in the case of organic aerosol analysis, a lot of the databases simply do not have entries for common substances contained in organic aerosol. Thoma et al. identified the problem and started to create a database targeted towards organic aerosol [19].

#### Creation of quasi-isolated MS2 spectra and differences in ion intensity

**Fig. 4:**
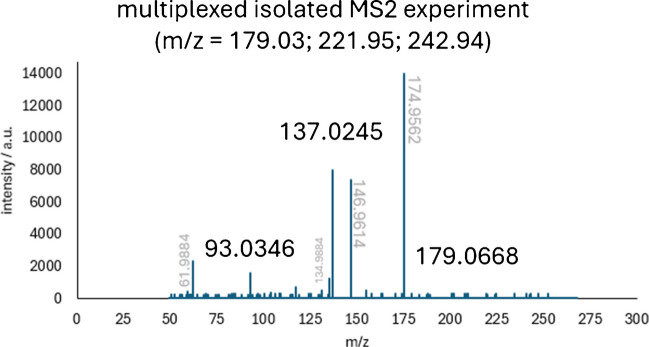
Isolated MS^2^ spectrum of acetylsalicylic acid as captured in a multiplexed dd-MS experiment with other m/z of: 221.95 and 242.94. In gray are the masses of unrelated fragments and in black are the masses of related fragments

**Fig. 5:**
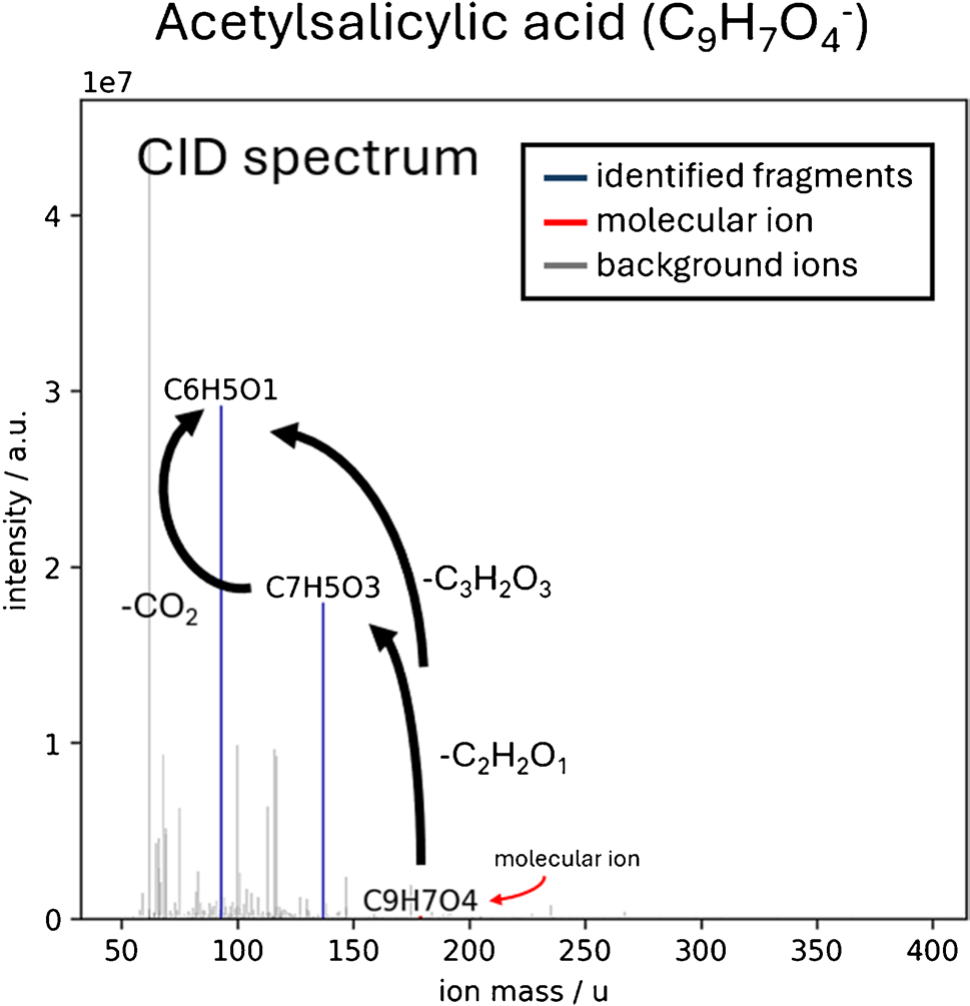
Quasi-isolated MS^2^ spectrum of acetylsalicylic acid (C_9_H_7_O_4_^–^, red) as captured during the standard measurement with DIA and computed with the algorithm. The fragments (C_7_H_5_O_3_^–^ and C_6_H_5_O_1_^–^ ) of the precursor ion are highlighted in blue. All other m/z ratios that were measured in the AIF spectrum are shown in light gray. The arrows annotate the corresponding neutral losses

If fragments are identified, a quasi-isolated MS^2^ spectrum can be created as is shown in Fig. 5. In contrast to the originally captured AIF spectrum, this quasi-isolated MS^2^ spectrum looks significantly less crowded and is therefore human readable again. The standard measurements conducted in this work have shown that the algorithm is sufficiently powerful to create meaningful quasi-isolated MS^2^ spectra. This makes the transition from traditional DDA experiments with isolated MS^2^ spectra to DIA experiments easier for the experimenter, as the resulting mass spectra look nearly identical. A direct comparison of a traditional DDA MS^2^ spectrum and a DIA quasi-isolated MS^2^ spectrum for acetylsalicylic acid is shown in Fig. 4 and Fig. 5.

As can be seen in the example, the overall intensity is significantly higher in the AIF spectrum, which explains why some fragments are only visible in the AIF spectrum, but not in the dd-MS^2^ spectrum (e.g., 2-Methylsuccinic acid, 2-Nitrobenzoic acid, or 4-Methyl-2-nitrophenol in Table 2). This can be explained by the significantly higher intensity in the AIF spectrum than in the dd-MS2 spectrum. To capture an isolated MS^2^ spectrum, the quadrupole needs to filter out all ions, except for the selected mass. This process is inherently inefficient, and the transmission efficiency at the selected m/z value is affected by the window size of the bandpass filter. This is a huge advantage of the full MS/AIF method over dd-MS^2^ measurements, as more fragments and therefore more structural information can be obtained in a single experiment. Especially for future applications where possible fragments are computed to elucidate the structure of the precursor ion, more fragments should yield better structural approximations.

#### Problem coelution

A problem that does not exist when isolated MS^2^ experiments are conducted is the coelution of multiple components at the same retention time. If two or more compounds are eluted at the same time, possess a similar peak shape, and have a similar formula, the algorithm is not able to keep the different molecules and fragments apart, as both precursor ions are fragmented at the same time and therefore fragments of both molecules are present in the captured AIF spectrum. The algorithm then tries to match the measured fragments with the corresponding precursor ion, but as there are two possible precursor ions, it is not able to distinguish the fragments and instead matches all the fragments to both precursor ions. This shows the necessity to have good chromatographic separation to minimize the number of coelutions with similar peak shapes.

During routine internal use of the tool with “real” aerosol samples, we found that the problem described above is not as pronounced as expected, and a series of random checks yielded only plausible fragments. A more detailed analysis of an environmental aerosol filter sample analyzed with the presented tool can be found in the supplementary information. The results are consistent with other analyses and provide the expected results. The Van Krevelen diagram created from the analysis results shows that the majority of compounds are within plausible heuristic limits, and the diagram for the oxidation state of carbon shows the expected increase in compound numbers at lower C atom numbers and an increasing O/C ratio.

### Software/data processing

**Fig. 6:**
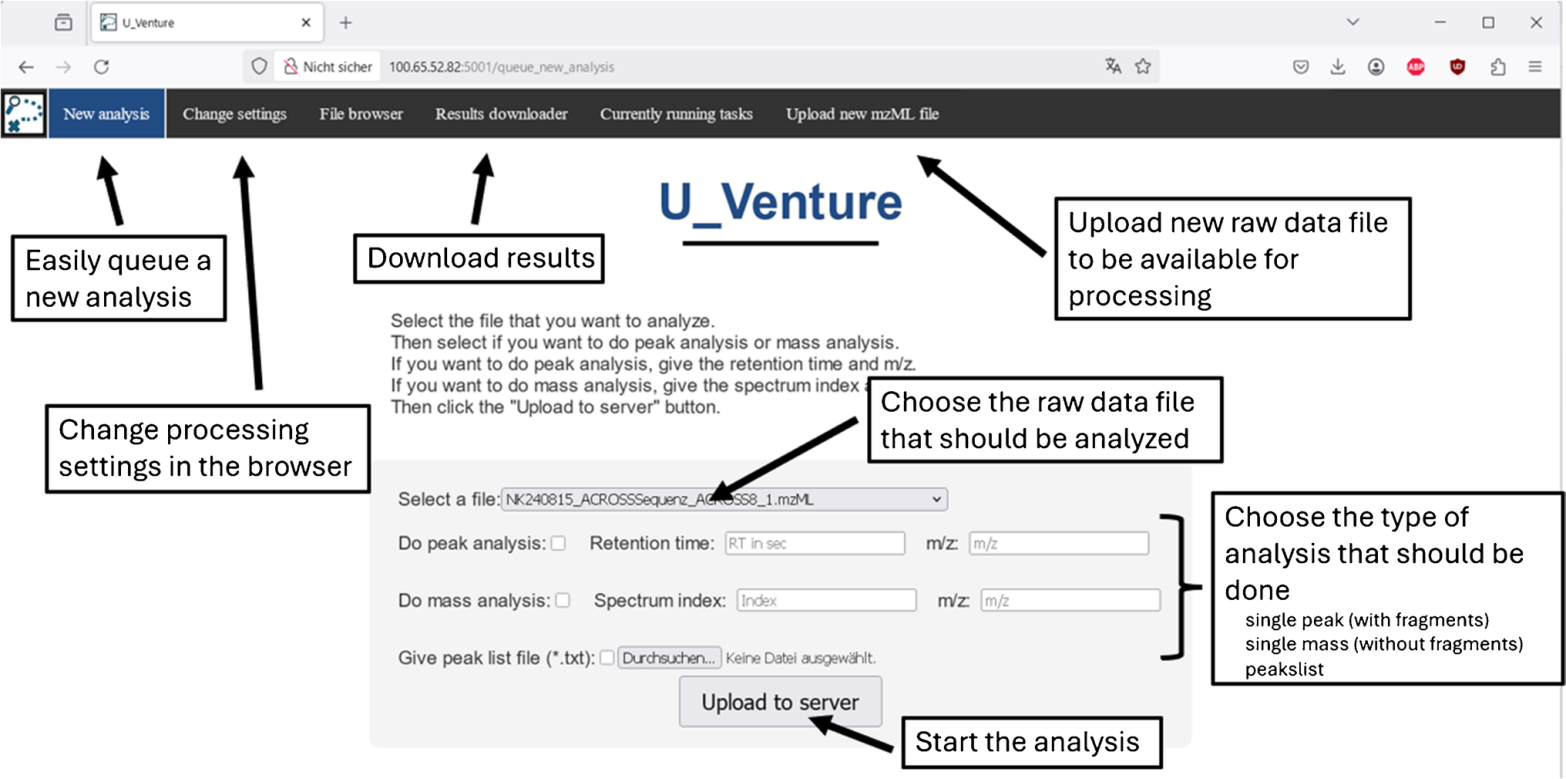
User interface of the website. New analysis runs can easily be started by selecting the appropriate raw data and selecting the type of analysis

To minimize entry hurdles, a website acts as the interface between the tool itself and the user (Fig. 6). If the tool is implemented in a mass spectrometry laboratory, it can be installed on a local server which is hosting the website, which can then be accessed by anyone attached to the local network. This allows the end user to access the website via a web browser without the need for any manual installation on the client side. Everything is managed on the local server, making maintenance easy and reducing costs, as only one powerful computer (acting as the server) needs to be bought, in contrast to many powerful computers — one for every end user.

To ensure reliable predictions, it is necessary to adjust certain parameters and thresholds of the tool to fit with the specific instrument that is used for the analysis. However, these settings need to be configured only once before starting the actual analyses. Tests have shown that once the correct tool settings for the individual MS instrument and MS instrument method have been found, the tool gives reliable results, even when other parameters like the LC method change. Therefore, once properly configured, the tool can be used by all members of a team, independently of their individual research focus and without any additional setup. This highlights the user-friendliness of the tool, which was also one of the goals of this development.

To submit an analysis, the user uploads the raw MS data as a.mzML file (open-source raw data file format for MS files) on the website. The server then redirects to the homepage where the user needs to provide information about the molecular ions (m/z and retention time) to be analyzed, and the tool then automatically starts the analysis. If other analyses are currently running, the new analysis will be queued and analyzed in order of their submission.

Once completed, the user can obtain the results as a downloadable *.zip* file.

### Summary

The results show that the presented tool delivers comparable results to traditional/manual analysis while significantly reducing the time needed to analyze the sample. DIA is not biased in any direction, provides higher fragment ion abundances, and therefore better limits of detection (LOD), contains all the information that is contained within the sample, and should therefore be preferred over DDA. For the instruments and methods used in this work, the decreased time resolution of the full MS spectra did not have adverse effects on the traditional evaluation of the full MS spectra. Therefore, we recommend that even if the AIF spectra contained in the measurement are currently not evaluated, one should still measure samples with the data-independent acquisition mode full MS/AIF measurements.

## Supplementary Information

Below is the link to the electronic supplementary material.

Supplementary Material 1 (DOCX 428 KB)

Supplementary Material 2 (TXT 55.0 KB)

## Data Availability

The source code and sample data are available at 10.5281/zenodo.16272009 and at https://github.com/NKa1409/UVenture.
