# Supplementary material for: CID fragment annotation from data-independent experiments in non-target organic aerosol analysis: presenting an easy-to-use tool
Source: Anal Bioanal Chem. 2025 Nov 18;417(30):6961–71. doi: 10.1007/s00216-025-06184-5 (PMC12680884; doi:10.1007/s00216-025-06184-5)
Supplement: Supplementary file 1 — Supplementary Material 1 (DOCX 428 KB) [file 216_2025_6184_MOESM1_ESM.docx]

Supporting Information for:

CID fragment annotation from data-independent experiments in non-target organic aerosol analysis: presenting an easy-to-use tool

Niklas Karbach^1^, Thorsten Hoffmann^1^✉

✉: t.hoffmann@uni-mainz.de

# S1

To better show the functionality of the presented tool for the analysis of “real” samples, an aerosol filter sample taken during the ACROSS campaign (summer 2022, approx. 40 km south-west of Paris) was analyzed. The filter contained many individual compounds from both anthropogenic and biogenic sources. The filter was extracted according to the method described in [4], measured, and was then analyzed by the tool. Retention times and masses of individual peaks were determined by mzMine4 before then being passed to the tool for prediction of the molecular formula and identification of fragments. However, in a recently published version of the presented tool (see latest version on Github), a peak detection algorithm is already implemented within the tool, and therefore all steps necessary for the analysis can be performed solely with the presented tool.


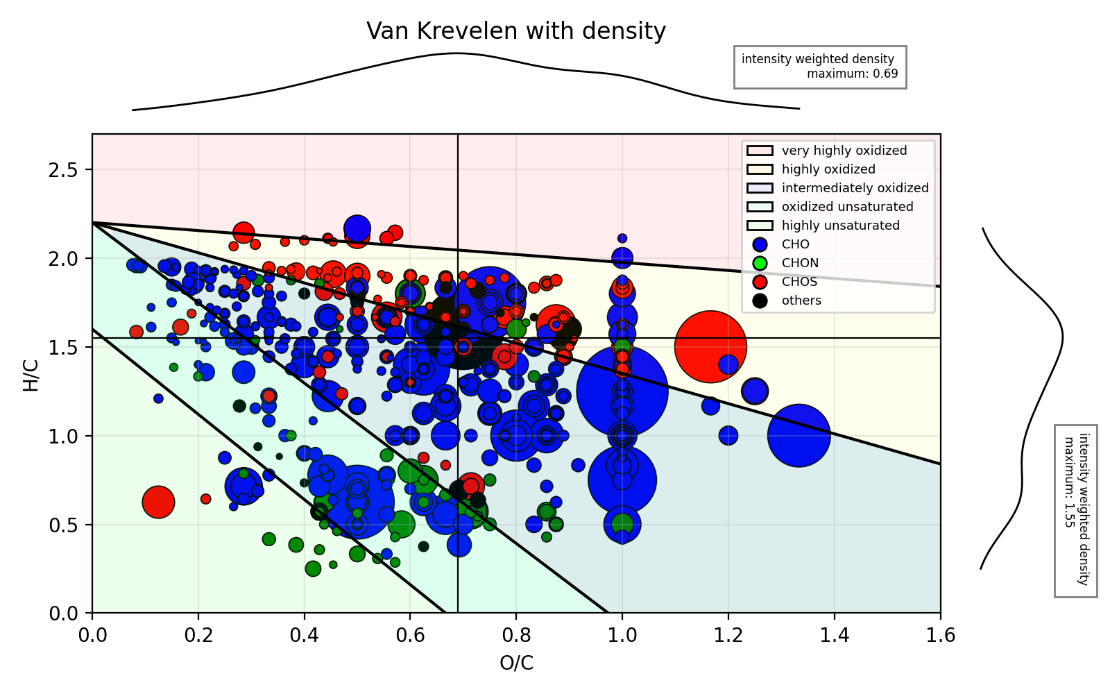


Figure 1: Van Krevelen Plot of the "real" aerosol filter sample. The color of the points corresponds to the atomic subset of the individual compounds, and the size corresponds to the measured intensity. The shaded areas highlight zones of similar oxidation state (see[1]).


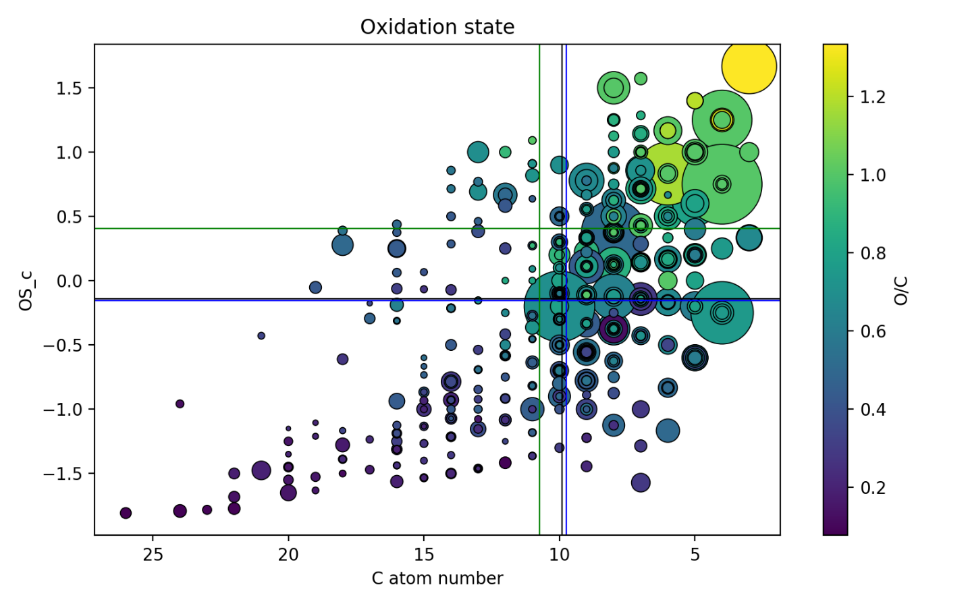


Figure 2: Kroll Plot of the calculated carbon oxidation state (OS_c), see [2], of the identified molecular ions plotted against the carbon atom number. The color corresponds to the individual O/C ratio of the precursor ion. As expected, the OS_c increases with decreasing carbon atom number (see [2, 3]).

The Van Krevelen plot (Figure 1) looks similar to other VK plots of ambient aerosol filters reported in literature, indicating that the tool delivers correct results. This assumption is further supported by the carbon carbon oxidation state plot shown in Figure 2. Here, the typical increase of OS_c with decreasing carbon atom numbers can be seen. This effect can easily be explained by the shift from functionalization towards fragmentation as compounds are atmospherically oxidized. This has previously been reported for “real” samples, as well as chamber studies [2, 5-7].

To keep the document easily readable only the 20 most intense compounds of the real sample are shown here in Table 1. All of the 595 identified compounds of the “real” sample are shown in the supporting textfile. The predicted formula is given as [M-H]^-^.

Table 1: Selection of the 20 most intense compounds that were identified with the presented tool. The predicted formula is given as [M-H]^-^.

| RT | m/z | Pred. formula | Formula score | Intensity | Fragments | Neutral losses |
| --- | --- | --- | --- | --- | --- | --- |
| 232.2 | 117.0188 | C4H5O4 | 38.1 | 37956069 | 'C2H3O3' | 'C2H2O1' |
| 471.1 | 294.0652 | C10H16N1O7S1 | 675.7 | 329771527 | 'O4S1', 'C10H15O5S1', 'C9H16O4S1', 'C9H16O6S1' | 'C10H16N1O3', 'H1N1O2', 'C1N1O3', 'C1N1O1' |
| 282.1 | 165.0188 | C8H5O4 | 514.2 | 258334392 | 'C7H5O2' | 'C1O2' |
| 405.6 | 225.0070 | C6H9O7S1 | 215.2 | 242312454 | 'C6H9O4', 'C5H7O2', 'C5H5O1', 'C6H7O3' | 'O3S1', 'C1H2O5S1', 'C1H4O6S1', 'H2O4S1' |
| 420.2 | 103.0396 | C4H7O3 | 328.1 | 237837994 | 'C3H5O1', 'C3H3O3', 'C2H1O3', 'C2H3O2', 'C4H5O2', 'C3H3O2', 'C4H7O2', 'C4H5O1', 'C4H3O2' | 'C1H2O2', 'C1H4',  'C2H6', 'C2H4O1', 'H2O1', 'C1H4O1',  'O1',  'H2O2',  'H4O1' |
| 505.1 | 294.0653 | C10H16N1O7S1 | 657.3 | 224597472 | 'N1O3', 'C7H7O1', 'C8H7O3' | 'C10H16O4S1', 'C3H9N1O6S1', 'C2H9N1O4S1' |
| 291.8 | 115.0031 | C4H3O4 | 406.8 | 209815043 | 'C3H3O2' | 'C1O2' |
| 165.4 | 103.0031 | C3H3O4 | 217.3 | 167533860 | 'C2H3O2' | 'C1O2' |
| 89.0 | 187.0608 | C8H11O5 | 470.1 | 102048287 | 'C7H9O2', 'C6H7O3' | 'C1H2O3', 'C2H4O2' |
| 278.5 | 129.0188 | C5H5O4 | 282.2 | 96881774 | 'C4H5O2', 'C3H3O3' | 'C1O2', 'C2H2O1' |
| 330.9 | 179.0345 | C9H7O4 | 436.7 | 50432902 | 'C8H7O2', 'C7H7' | 'C1O2', 'C2O4' |
| 352.9 | 209.0088 | C9H5O6 | 399.3 | 50194712 | 'C7H5O2', 'C8H5O4' | 'C2O4', 'C1O2' |
| 474.7 | 253.0387 | C8H13O7S1 | 500.4 | 50103274 | 'C8H13O4', 'C7H11O2' | 'O3S1', 'C1H2O5S1' |
| 282.1 | 121.0290 | C7H5O2 | 140.7 | 42079495 | 'None' | 'None' |
| 539.3 | 227.9894 | C8H4O8 | 24.1 | 41498579 | 'None' | 'None' |
| 68.0 | 189.0765 | C8H13O5 | 321.0 | 41140290 | 'None' | 'None' |
| 232.2 | 117.0188 | C4H5O4 | 38.2 | 37956070 | 'C2H3O3' | 'C2H2O1' |
| 571.0 | 182.0091 | C7H4N1O5 | 99.1 | 33919524 | 'None' | 'None' |
| 278.5 | 187.0244 | C7H7O6 | 358.8 | 33709508 | 'C4H5O2', 'C6H5O3', 'C3H3O3' | 'C3H2O4', 'C1H2O3', 'C4H4O3' |
| 61.9 | 115.0396 | C5H7O3 | 152.6 | 32107670 | 'C3H5O2', 'C4H5O2', 'C5H5O2', 'C4H3O3', 'C4H5O1' | 'C2H2O1', 'C1H2O1', 'H2O1',  'C1H4', 'C1H2O2' |

# References

1. Zhang Y, Wang K, Tong H, Huang R-J, Hoffmann T.: The maximum carbonyl ratio (MCR) as a new index for the structural classification of secondary organic aerosol components. Rapid Commun Mass Spectrom. 2021; <https://doi.org/10.1002/rcm.9113>
2. Kroll, J., Donahue, N., Jimenez, J. et al.: Carbon oxidation state as a metric for describing the chemistry of atmospheric organic aerosol. Nature Chem 3. 2011;. <https://doi.org/10.1038/nchem.948>
3. Thoma, M., Bachmeier, F., Gottwald, F. L., Simon, M., and Vogel, A. L.: Mass spectrometry-based Aerosolomics: a new approach to resolve sources, composition, and partitioning of secondary organic aerosol, Atmos. Meas. Tech. 2022; <https://doi.org/10.5194/amt-15-7137-2022>
4. Leppla, D., Zannoni, N., Kremper, L., Williams, J., Pöhlker, C., Sá, M., Solci, M. C., and Hoffmann, T.: Varying chiral ratio of pinic acid enantiomers above the Amazon rainforest. Atmos. Chem. Phys. 2023; <https://doi.org/10.5194/acp-23-809-2023>
5. Kroll, J., Lim, C., Kessler, S., Wildon, K.: Heterogeneous Oxidation of Atmospheric Organic Aerosol: Kinetics of Changes to the Amount and Oxidation State of Particle-Phase Organic Carbon. J. Phys. Chem. A. 2015; <https://doi.org/10.1021/acs.jpca.5b06946>
6. Zhang, Y., Gao, X., Hou, X., Liu, M., Han, J.: Zhang, H. Chemical Characterization of Rural Organic Aerosol in the North China Plain Using Ultrahigh-Resolution Mass Spectrometry. Atmosphere. 2023; <https://doi.org/10.3390/atmos14111636>
7. Kourtchev, I., Godoi, R. H. M., Connors, S., Levine, J. G., Archibald, A. T., Godoi, A. F. L., Paralovo, S. L., Barbosa, C. G. G., Souza, R. A. F., Manzi, A. O., Seco, R., Sjostedt, S., Park, J.-H., Guenther, A., Kim, S., Smith, J., Martin, S. T., and Kalberer, M.: Molecular composition of organic aerosols in central Amazonia: an ultra-high-resolution mass spectrometry study. Atmos. Chem. Phys. 2016; <https://doi.org/10.5194/acp-16-11899-2016>
